# The Role of Autophagy in Liver Diseases: Mechanisms and Potential Therapeutic Targets

**DOI:** 10.1155/2015/480508

**Published:** 2015-03-19

**Authors:** Raffaele Cursio, Pascal Colosetti, Patrice Codogno, Ana Maria Cuervo, Han-Ming Shen

**Affiliations:** ^1^Service de Chirurgie Digestive et Transplantation Hépatique, Hôpital l'Archet 2, Université de Nice Sophia Antipolis, 06202 Nice, France; ^2^Inserm U1065-C3M, Equipe 2, Université de Nice Sophia Antipolis, 06204 Nice, France; ^3^Inserm U1151-CNRS UMR 8253, Institut Necker Enfants-Malades, Université Paris Descartes-Sorbonne Paris Cité, 75014 Paris, France; ^4^Department of Developmental and Molecular Biology, Institute for Aging Research, Albert Einstein College of Medicine, Bronx, NY 10461, USA; ^5^Department of Physiology, Yong Loo Lin School of Medicine, National University of Singapore, Singapore 117597

Autophagy, or cellular self-digestion, is an orchestrated cellular pathway crucial for development, differentiation, homeostasis, and survival of cells. The autophagic process is used to eliminate unwanted proteins and damaged organelles and to remove intracellular microbial pathogens. Normal liver function requires hepatocellular basal autophagy. In fact, due to their high biosynthetic activity and role in protein turnover and carbohydrate storage, hepatocytes may be particularly dependent on basal autophagy for their normal physiological functions.

Autophagy seems to play an important role not only in normal liver physiology, but also in the pathogenesis of liver diseases such as nonalcoholic and alcoholic fatty liver, drug-induced liver injury, protein conformational liver diseases, viral hepatitis, fibrosis, aging, liver cancer, and liver ischemia-reperfusion injury.

In this special issue, original research and review articles have focused on the role of autophagy in the pathogenesis of the above mentioned liver diseases, bringing new knowledge and suggesting modulation of autophagy as basis for possible treatments for these pathologies.

The liver is one of the principle regulators of lipid in the body. Obesity is closely associated with an increased risk of metabolic diseases including nonalcoholic fatty liver disease (NAFLD). NAFLD ranges from isolated steatosis to nonalcoholic steatohepatitis (NASH) and steatofibrosis, which sometimes leads to cirrhosis and to hepatocellular carcinoma (HCC). Under physiological conditions, autophagy participates in the basal turnover of lipids by engulfing and degrading lipid droplets. In obesity, a decrease of autophagy levels in hepatocytes has been described. As illustrated by V. J. Lavallard and P. Gual in their review, several mechanisms may account for this decline. The authors describe the role of autophagy in specific cells, including hepatocytes, macrophages, hepatic stellate cells (HSCs), and cancer cells and outline its role in the evolution of hepatic complications associated with obesity, from steatosis to HCC. They suggest that activation of autophagy in hepatocytes could constitute a therapeutic approach against hepatic complications of obesity.

In the pathogenesis of NASH, the mitochondrial dysfunction participates in different levels since it impairs fatty liver homeostasis and induces overproduction of reactive oxygen species (ROS) that in turn triggers lipid peroxidation, cytokines release, and cell death. Mitochondrial uncoupling protein 2 (UCP2) seems to have a role in the development of NASH. In their original study J. Lou et al. investigate the relationship between UCP2 and hepatoma cells autophagy in palmitic acid- (PA-) induced lipotoxicity. They provide evidence that increasing UCP2 expression in hepatoma cells contributes to autophagy. Moreover, UCP2 is a proliferative factor that also has an antiapoptotic role during PA-induced liver injury. These results provide insights into potential NASH therapies.

As outlined in the review of Y. Li et al., autophagy plays significant roles in preserving hepatocyte homeostasis and viability in alcohol consumption-induced multiple tissue/organ injuries including hepatic steatosis and liver injury, pancreatitis, impaired heart function, brain damage, and loss of muscle mass. Although autophagy serves as a cellular protective mechanism against alcohol-induced tissue injury in most tissues, it can be detrimental in heart and muscle. In the liver, it seems that alcohol metabolism through alcohol dehydrogenase and cytochrome P450 family 2, subfamily E, polypeptide 1 (Cyp2E1), is required for autophagy activation. Acute alcohol treatment also induces forkhead box-containing protein class O (FoxO) family of DAF-16 like transcription factor 3-mediated autophagy. Finally, autophagy seems to selectively remove damaged mitochondria and excess lipid droplets and in turn attenuates alcohol-induced steatosis and liver injury.

Liver fibrosis is a common wound healing response to chronic liver injury of all causes. Its end-stage cirrhosis is responsible for high morbidity and mortality worldwide. As a multifaceted partner in liver fibrosis, autophagy elicits divergent and cell-specific effects during chronic liver injury as outlined in the review by A. Mallat et al. In fact, autophagy enhances fibrogenic properties in HSCs and liver fibrosis. In contrast, through its anti-inflammatory effects in macrophages and hepatoprotective effects in hepatocytes, autophagy limits the development of liver fibrosis.

The dual role played by autophagy in inflammation and lipid metabolism in hepatitis C virus- (HCV-) infected liver cells raises an important question regarding the contribution of autophagy defects in disease progression towards steatohepatitis, fibrosis, cirrhosis, and HCC in patients with chronic HCV infection. In their review, T. Vescovo et al. discuss the molecular mechanisms that link the HCV life cycle with the autophagy machinery. In particular, the authors outline the role of HCV/autophagy interaction in dysregulating inflammation and lipid homeostasis and the potential applications in the treatment of HCV-infected patients.

Alpha-1-antitrypsin deficiency (ATD) is one of the most common genetic causes of liver disease. ATD is the prototype of liver disease caused by pathologic accumulation of aggregated mutant alpha-1-antitrypsin Z (ATZ) within liver cells. Accumulation of ATZ in the liver specifically activates autophagy. In their review, A. S. Chu et al. summarize research advances in autophagy and genetic liver diseases. They discuss autophagy enhancing strategies for liver disease due to ATD and to other genetic liver diseases, for example, inherited hypofibrinogenemia, caused by the proteotoxic effects of a misfolded protein. On the basis of recent evidence that autophagy plays a role in cellular lipid degradation, the authors also speculate about autophagy enhancing strategies for treatment of hepatic lipid storage diseases such as cholesterol ester storage disease.

As outlined by M. Kheloufi et al. in their review, increasing evidence demonstrates that autophagy plays a critical role in acute liver injury related to severe anorexia nervosa (AN) and to drug overdose. AN has the highest rate of mortality among eating disorders and can be associated with severe liver insufficiency. Overdose of acetaminophen (APAP), a widely used antipyretic and analgesic drug, is the first cause of acute liver failure in humans. Efavirenz, a nonnucleoside reverse transcriptase inhibitor widely used to treat human immunodeficiency virus (HIV) infections, can be hepatotoxic in some patients. Increased liver autophagy levels are a common feature of these disorders. Autophagy is mainly hepatoprotective. In AN, during the first phase of weight loss, liver blood test abnormalities are moderate suggesting that autophagy can cope with nutrient deprivation. During that period, autophagy is protective and prevents cell death. When starvation continues and body mass index reaches a critically low level, excessive activation of autophagy leads to hepatocyte cell death and liver insufficiency. After APAP or Efavirenz exposure, autophagy removes damaged mitochondria, and liver injury appears only when this process is either blocked by other factors or overwhelmed.

Liver ischemia-reperfusion (I-R) injury which occurs during liver resection, liver transplantation, and hemorrhagic shock can induce liver dysfunction and can increase patient morbidity and mortality after liver surgery, particularly liver transplantation and hemorrhagic shock. Whether autophagy protects from or promotes liver injury following warm and/or cold I-R remains to be further elucidated. R. Cursio et al. summarize in their review the current knowledge on liver I-R injury focusing on both the beneficial and detrimental effects of liver autophagy following warm and/or cold liver I-R. The autophagic cell response to warm and/or cold liver I-R may delay apoptosis and necrosis and thus ultimately increases the possibility for novel therapeutic intervention to diminish the extent of warm/cold liver I-R injury.

The liver is an organ of great complexity with multiple types of cells, parenchymal and nonparenchymal, and with multiple functions too. Dysregulation of liver autophagy functions has an impact on liver physiology, but also on pathologies of the liver. Dysregulation or decrease of autophagy in alcoholic liver diseases can lead to liver cell death, steatohepatitis, and HCC. In liver I-R injury, autophagy has mainly prosurvival effects, while, in hepatitis C infection, increased autophagy supports virus replication. In some diseases, autophagy may have opposite effects; for example, in liver fibrosis, autophagy may be profibrotic as well as antifibrotic. This dual role played by autophagy is one of the main challenges for the establishment of future therapeutic approaches to these liver diseases.

*Raffaele Cursio* *Pascal Colosetti* *Patrice Codogno* *Ana Maria Cuervo* *Han-Ming Shen*

